# The gut microbiota influences neurodegenerative diseases through the gut-brain axis: molecular mechanisms and effects on immune function

**DOI:** 10.3389/fimmu.2025.1739329

**Published:** 2026-01-12

**Authors:** Jiaheng Yang, Xiangzhun Song, Su Yan, Qianxun Li, Wenying Yang

**Affiliations:** 1 Clinical College of Medicine, Changchun University of Chinese Medicine, Changchun, China; 2 Department of Gastroenterology, Jilin Province People’s Hospital, Changchun, Jilin, China

**Keywords:** AD, fecal microbiota transplantation, gut-microbiota-brain axis, NDDs, PD

## Abstract

The pathogenesis of neurodegenerative diseases (NDDs), including Alzheimer’s disease (AD), Parkinson’s disease (PD), and amyotrophic lateral sclerosis (ALS), is complex and multifactorial. Recent studies indicate that the microbiota-gut-brain axis (MGBA) plays a crucial role in the development and progression of NDDs. The MGBA concept reveals a complex bidirectional regulatory network between the gut microbiota and the central nervous system (CNS), linking them through immune, neural, endocrine, and metabolic pathways. This review summarizes the components of the MGBA, communication pathways between gut microbiota and the brain, and mechanisms by which gut microbiota influence the onset and progression of NDDs. Finally, preclinical therapeutic approaches for NDDs are discussed, evaluating preclinical trial data for probiotics, prebiotics, and fecal microbiota transplantation.

## 1 Introduction

Neurodegenerative diseases (NDDs) represent a complex class of disorders characterized by progressive loss of neuronal function or structure, including Alzheimer’s disease (AD), Parkinson’s disease (PD), and amyotrophic lateral sclerosis (ALS) (1). A common feature of these diseases is protein misfolding and aggregation, leading to neuronal death and dysfunction, which in turn causes impairment of cognitive, motor, or autonomic functions (2). In recent years, with the acceleration of global aging, the incidence of NDDs has significantly increased, becoming a major social and economic burden. Although traditional research has made some progress in the pathological mechanisms and therapeutic strategies of NDDs, their complex etiology and multifactorial interactions remain incompletely elucidated. The pathogenesis of NDDs involves multiple pathways, including pathological protein aggregation, synaptic dysfunction, protein homeostasis imbalance, mitochondrial dysfunction, neuroinflammation, ferroptosis, and interactions between genetic and environmental factors (3–5). With advances in biology, the role of gut microbiota in NDDs has garnered widespread attention. Research indicates that gut microbiota influence brain function through multiple pathways, including neural, endocrine, immune, and metabolic systems. Their dysregulation is closely associated with the pathological processes of NDDs such as AD and PD (6). Specifically, the gut microbiota communicates bidirectionally with the CNS via the gut-brain axis (GBA), a mechanism involving multifaceted regulation of endocrine, immune, and neural signaling (7).

The Microbiota-Gut-Brain Axis (MGBA) theory reveals a complex bidirectional regulatory network between the gut microbiota and the CNS, offering new insights into the pathological mechanisms of NDDs (8). Dysregulation of the MGBA is considered a key component in the pathogenesis of NDDs. Research indicates that the gut microbiota directly influences CNS function and homeostasis by modulating immune responses, neurotransmitter synthesis, metabolite production, and blood-brain barrier (BBB) permeability (9). For example, short-chain fatty acids (SCFAs) and neuroactive metabolites (e.g., 5-Hydroxytryptamine and dopamine) produced by the gut microbiota can be transported to the brain via the GBA, regulating neuronal activity and microglial function (10). Furthermore, gut microbiota dysbiosis (i.e., gut microbial imbalance) can induce systemic inflammation and oxidative stress, thereby promoting the pathological progression of NDDs (11, 12). Research indicates that dysbiosis of the gut microbiota may compromise the integrity of the BBB by inducing systemic chronic inflammation (SCI) and abnormal immune responses, ultimately leading to neuroinflammation and neurodegeneration (13, 14).

Therefore, this paper introduces the gut microbiota and its metabolites, and reviews the effects of gut microbes on the nervous system via the GBA. Subsequently, it highlights the effects of gut microbiota on the immune system in NDDs such as AD, PD, and ALS, and explores interventions targeting the gut microbiota as potential therapeutic targets for NDDs. This aims to enhance understanding of the pathogenesis of NDDs and provide new targets and therapeutic approaches for their treatment.

## 2 Gut microbiota-gut-brain axis

The core components of the MGBA include the gut microbiota, intestinal epithelial barrier, enteric nervous system (ENS), immune system, endocrine system, and CNS. It not only regulates digestive system functions but also profoundly influences brain development and the onset and progression of various neurological disorders (15).

### 2.1 Components of the gut microbiota-gut-brain axis

The gut microbiota refers to the vast microbial community residing within the human gastrointestinal tract, comprising bacteria, viruses, fungi, and archaea, numbering in the trillions (16). These microorganisms not only participate in digestion, nutrient absorption, and vitamin synthesis but also interact with the host’s immune, nervous, and metabolic systems through complex signaling networks, forming the so-called “gut-microbiota-host axis” (17). Through symbiosis with the host, gut microbes interact via metabolites, neurotransmitters, and immune signals. Metabolic products of gut microbes include short-chain fatty acids (SCFAs), secondary bile acids, indole compounds, trimethylamine N-oxide (TMAO), and various gas molecules (e.g., H_2_S, NO, and CO) (18, 19). In addition to metabolic roles, the gut microbiota is essential for immune regulation. It guides the differentiation and function of immune cells—including T cells and dendritic cells-thereby upholding systemic immune homeostasis (20). Furthermore, by reinforcing the integrity of the intestinal epithelial barrier, the microbiota helps prevent translocation of pathogenic organisms and harmful agents, playing a critical role in host defense and overall health (21).

The intestinal epithelial barrier constitutes the primary physical and immunological line of defense, selectively restricting the passage of harmful agents into systemic circulation while actively communicating with the gut microbiota via chemosensory receptors and tight junction proteins (22). Its structural and functional integrity is essential for sustaining a stable host-microbiota relationship and preventing aberrant immune activation (23).

A second critical component is the ENS, a subdivision of the peripheral nervous system composed of intrinsic neurons and glial cells that autonomously regulate gut motility, secretion, and absorption (24). The ENS maintains bidirectional communication with the CNS through vagal and spinal pathways, forming a key neural relay within the MGBA (25). The CNS, encompassing the brain and spinal cord, receives and integrates signals from the gut through neural, endocrine, and immune pathways, thereby influencing behavior and cognitive function (26). Simultaneously, the gut-resident immune system engages in continuous interaction with commensal microbiota, deploying cytokines and inflammatory mediators that influence both local intestinal function and central nervous integrity. Dysregulation of this crosstalk has been associated with the pathophysiology of several neuropsychiatric conditions, including anxiety, depression, Major Depressive Disorder (MDD), and bipolar disorder (BD) (27, 28). Furthermore, systemic immune and endocrine pathways contribute to gut–brain signaling through circulating cytokines, hormones, and neuropeptides, collectively enabling multi-level communication across the MGBA (26).

### 2.2 Functions of the gut microbiota-gut-brain axis

The MGBA exerts broad influence on neurodevelopment, behavioral modulation, and disease susceptibility through multiple interconnected pathways. One primary mechanism involves the microbial production of signaling molecules—such as short-chain fatty acids (e.g., butyrate), neurotransmitters (e.g., serotonin and GABA), and indole-derived metabolites—that directly or indirectly shape neuronal plasticity and synaptic function (29). Research indicates that the gut microbiota influences serotonin synthesis by regulating the tryptophan metabolic pathway, thereby modulating mood and cognitive function (30).

Additionally, the gut microbiota regulates neuroinflammatory tone and neuroprotective mechanisms through vagus nerve activation and immune modulation. For example, in AD models, altered gut microbiota reduced neuroinflammation and cognitive impairment by increasing indole-3-acetic acid levels (31). The microbiota also contribute to neurodegenerative pathology by dynamically regulating BBB permeability, thereby controlling the CNS exposure to circulating metabolites and neurotransmitters (32).

When the composition of the gut microbiota changes-a condition known as dysbiosis-it may trigger a series of pathophysiological responses (33). This process promotes systemic immune activation (21, 34). Inflammatory signals are then relayed to the CNS via immune and neuroendocrine routes, ultimately driving neuroinflammation and neurodegeneration (33). Accumulating evidence underscores a strong association between gut microbiome dysbiosis and the pathogenesis of several neurodegenerative disorders, including AD, PD, and multiple sclerosis (MS) (35).

### 2.3 Core communication pathways

**Figure 1:**
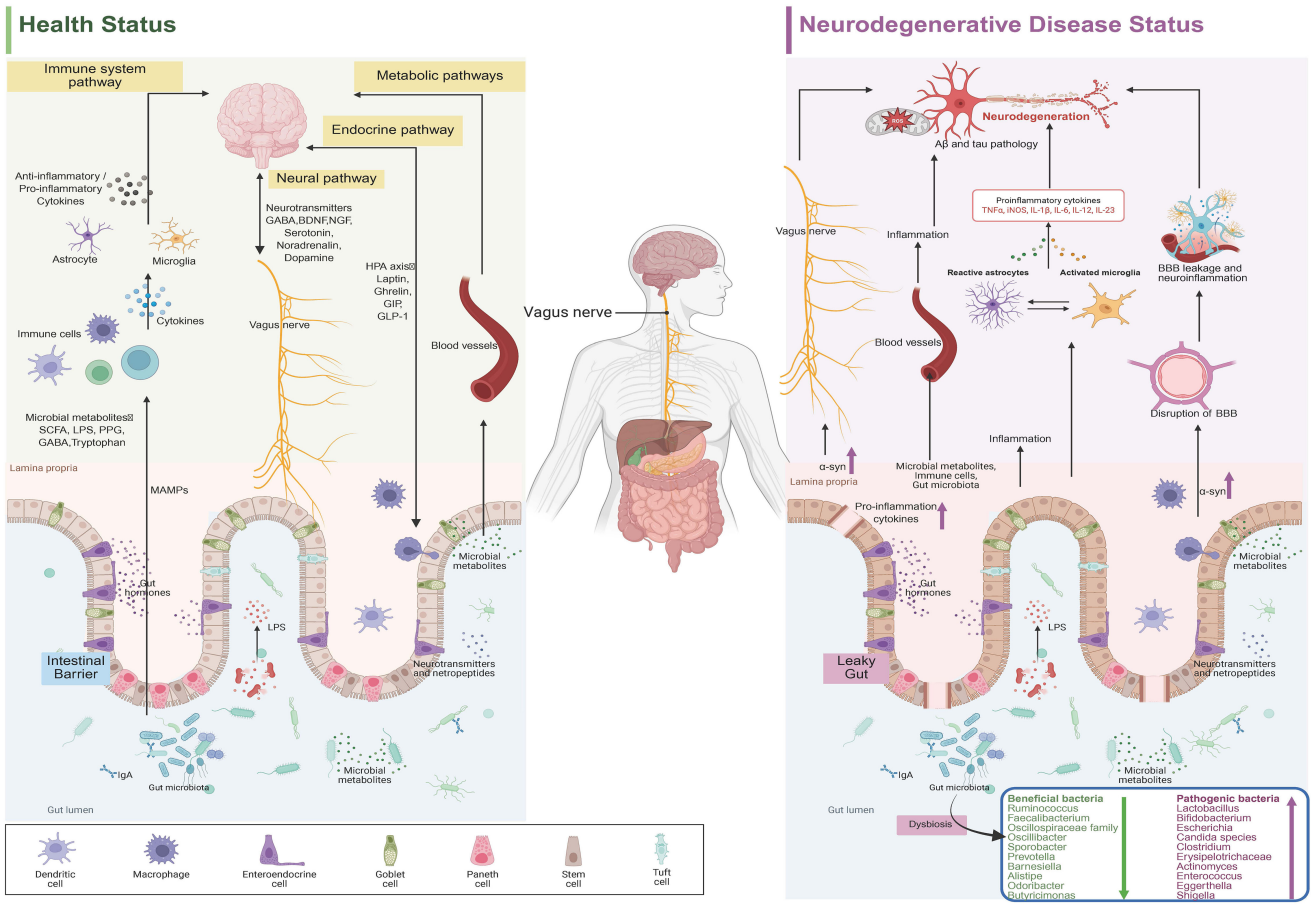
The communication pathway of gut microbiota-gut-brain axis in healthy status and the effect of gut microbial Dysbiosis on nerves in neurodegenerative diseases.

MGBA constitutes a complex bidirectional communication network that facilitates information exchange between gut microbiota and the CNS through multiple pathways (**Figure 1**). These core pathways include neural, immune, metabolic, and endocrine mechanisms, which together mediate gut-brain crosstalk.

#### 2.3.1 Neural pathways

Current research suggests that the neural pathways of the MGBA predominantly consist of the vagus nerve pathway, spinal cord afferent pathways, and local neural circuits mediated by the ENS (36). The vagus nerve serves as the most direct neural link between the gut and the brain. Gut microbes activate the vagus nerve via metabolites, thereby influencing brain functions including emotional regulation and cognitive performance (37). Additionally, the ENS—intrinsically organized with local neural networks—interacts with gut microbes and relays signals centrally, indirectly influencing brain activity and behavior (38). The spinal afferent pathways transmit mechanical and chemical stimuli from the distal gut, including the colon, to the central nervous system via dorsal root ganglion (DRG) neurons (39). The characteristic of the DRG whereby a single neuron innervates multiple targets allows the same sensory neuron to integrate heterogeneous signals from multiple levels of the intestine and affect visceral perception and cognitive function *via* the spinal cord - thalamus - cortex pathway (39, 40).

#### 2.3.2 Immune pathways

Gut microbes regulate the activity of intestinal immune cells to release cytokines. These cytokines affect BBB permeability and brain inflammatory states via the bloodstream (22). Furthermore, microbial metabolites are capable of directly shaping microglial maturation and function, thereby influencing neurodevelopmental and neuroinflammatory processes (16, 41).

#### 2.3.3 Metabolic pathways

Short-chain fatty acids (e.g., butyrate, propionate) produced by gut microbes fermenting dietary fiber reach the brain via the bloodstream, regulating neuroinflammation and neurotransmitter synthesis (8). Gut microbes also synthesize or modulate neurotransmitter precursors (e.g., tryptophan, 5-hydroxytryptophan), which subsequently influence central neurotransmission via humoral or neural routes (42).

#### 2.3.4 Endocrine pathways

Gut microbiota influence the hypothalamic-pituitary-adrenal (HPA) axis by regulating the secretion of gut peptides and hormones, thereby modulating stress responses and mood (43). Additionally, microbial metabolites such as bile acids and ammonia may alter BBB permeability, further enabling direct modulation of CNS function (44).

#### 2.3.5 Microbial metabolites

Gut microbes directly synthesize a range of neuroactive molecules, including gamma-aminobutyric acid, which can influence CNS activity upon reaching the brain via circulation or neural pathways (45). Additionally, bacterial structural components such as lipopolysaccharide (LPS) act as potent immunostimulants; their systemic translocation can provoke immune activation and sustained neuroinflammation, thereby accelerating the progression of NDDs (44).

#### 2.3.6 Barrier function

The integrity of the intestinal barrier function is fundamental to MGBA communication. Dysbiosis of gut microbiota can lead to impaired intestinal barrier function, known as “leaky gut.” Leaky gut allows bacteria and their metabolites to enter the systemic circulation via the bloodstream, subsequently affecting the integrity of the BBB, triggering neuroinflammation and neuronal damage (46). Disruption of these barriers is closely associated with various neurological disorders, including autism and AD (47). In AD research, gut dysbiosis is significantly correlated with intestinal permeability, which accelerates neuroinflammation and Aβ deposition through BBB disruption (48).

### 2.4 Gut microbiota dysbiosis in neuroinflammation and immune dysfunction

Gut microbiota dysbiosis is considered a key driver of neuroinflammation and immune dysfunction in NDDs (49). For example, mutations in the *GBA1* gene can induce microbial imbalance, which in turn exacerbates PD pathology through neuroimmune activation (50). Supporting this, preclinical studies show that microbiota depletion markedly reduces neuroinflammation and ameliorates motor deficits in disease models (51).

In NDDs, the gut microbiota influences the immune system through multiple pathways. First, gut microbiota directly shape the phenotype and function of immune cells (52, 53). Furthermore, the gut microbiota influences neuronal survival and function by modulating the activity of myeloid cells such as macrophages and microglia (54). These cells play critical roles in clearing neurotoxic substances like amyloid plaques and regulating neuroinflammation (55). Studies have demonstrated that microglia, as the primary immune effector cells in the central nervous system, display an over - activated phenotype under conditions of intestinal flora imbalance. This results in the release of a substantial quantity of pro - inflammatory factors (e.g., IL - 1β, TNF - α), which in turn leads to neuronal damage and the specific loss of dopaminergic neurons (56). For example, in AD, amyloid proteins secreted by the gut microbiota can cross-aggregate with Aβ in the brain *via* a molecular mimicry mechanism, which exacerbates neuroinflammation and synaptic damage (57, 58).

From an immunological perspective, dysbiosis of the gut microbiota can result in the impairment of intestinal barrier function, which facilitates the translocation of bacteria and their metabolic products (e.g., lipopolysaccharides) into the circulatory system. Subsequently, this activates peripheral immune cells and initiates a systemic inflammatory response. These inflammatory mediators can impact the central nervous system through multiple pathways. Firstly, the disruption of the integrity of the BBB enables cytokines and immune cells to infiltrate the brain parenchyma. Secondly, peripheral immune signals can be transmitted to the central nervous system via the vagus nerve or directly act on brainstem circumventricular organs (e.g., the area postrema) (59). For example, in PD, the age-associated decline in immune system function, combined with long-term exposure to environmental factors like pesticides or pathogens, can be further aggravated by gut microbiota dysbiosis, creating an ideal situation that facilitates the pathological spread of α-synuclein (49). It is noteworthy that the gut microbiota also influences the production of kynurenine through the regulation of the tryptophan metabolic pathway. This metabolic product can not only exert an effect on the enteric nervous system, leading to intestinal dysfunction, but also cross the BBB to directly modulate the immune balance of the central nervous system (50, 60).

The gut microbiota also modulates bile acid and tryptophan metabolism, with downstream effects on neurotransmitter synthesis and neuronal viability (61). Specific tryptophan metabolites such as indole-3-acetaldehyde (I3AA) have been shown to regulate TGFβ signaling and CD4^+^ T cell differentiation, thereby exacerbating intestinal and neuroinflammation (62).

Collectively, the gut microbiota influences the MGBA through multiple core communication pathways-encompassing neural, immune, metabolic, endocrine, microbial metabolite, and barrier functions. These pathways are highly interconnected, forming an integrated network that collectively regulates bidirectional gut–brain communication and ultimately shapes host physiology and disease susceptibility. A deeper understanding of these mechanisms will not only help clarify the pathogenesis of NDDs but also provide a scientific rationale for developing novel microbiota-targeted therapeutic strategies.

## 3 The role of the gut microbiota in neurodegenerative diseases

The MGBA, a bidirectional communication system, tightly links the gut microbiota with the CNS through neural, endocrine, and immune pathways, thereby playing a pivotal role in the pathogenesis of NDDs (31, 35, 63).

### 3.1 Alzheimer’s disease

As a NDDs, AD involves a complex pathogenesis encompassing multiple factors such as chronic neuroinflammation, Aβ, and abnormal tau phosphorylation. In AD, the gut microbiota influences disease progression by regulating Aβ deposition and tau hyperphosphorylation (55, 64). Gut microbiota dysbiosis has been demonstrated to be closely associated with AD pathogenesis. In AD patients, the abundance of Firmicutes and Bifidobacteria in the gut is reduced, while the abundance of Bacteroidetes and Proteobacteria is increased (65). This imbalance may induce “Leaky Gut” syndrome, allowing pathogen-associated molecular patterns (PAMPs) such as LPS to enter the bloodstream and trigger systemic low-grade inflammation (66). Research indicates that gut dysbiosis-induced intestinal permeability facilitates bacterial LPS and Aβ entry into the bloodstream, further activating microglia and exacerbating AD-associated neuroinflammation (67). Microglia, the primary immune cells of the CNS, exhibit dysfunction that constitutes a core pathological feature of AD. Certain gut microbial metabolites may induce abnormal activation of microglia and cross-seeding of amyloid through molecular mimicry mechanisms, further exacerbating AD pathology (68). Additionally, gut dysbiosis can cause astrocyte dysfunction, thereby impairing neuronal energy metabolism and antioxidant capacity, promoting neuronal degeneration (20). Microbial metabolites-including SCFAs, LPS, and trimethylamine N-oxide (TMAO)-also contribute to AD progression by enhancing systemic inflammation, disturbing cerebral Aβ clearance, and directly activating microglia, which intensifies neuroinflammation and neuronal injury (69–72). Moreover, gut microbes influence cognitive function in AD through the regulation of glutamate metabolism. Specific bacteria, such as *Bacteroides vulgatus* and *Campylobacter jejuni*, can lower levels of the glutamate metabolite 2-ketoglutarate, potentially altering NMDA receptor function and synaptic plasticity (73). Other microbiota-derived molecules, including secondary bile acids and tryptophan metabolites, may also modulate brain function and immune activity, thereby shaping AD trajectory (74).

Additionally, the interaction between the gut microbiota and the immune system constitutes a key mechanism in the onset and progression of AD. Research indicates that the efficacy of AD immunotherapies may partially depend on the regulatory role of the gut microbiota. For example, in AD mouse models, tau-targeted immunotherapy significantly alters the composition of the gut microbiota, thereby influencing treatment outcomes (75).

### 3.2 Parkinson’s disease

Dysbiosis of the gut microbiota has been demonstrated to be closely associated with PD. In the research on biodiversity, the Simpson’s Index and the Species Evenness Index are frequently employed for assessment (76). Specifically, the Simpson’s Index is utilized to quantify the concentration or dominance within a community (77), while the Species Evenness Index is designed to measure the uniformity of the distribution of individual numbers of various species within a community (78). Studies report reduced Simpson and species evenness indices in PD patients, reflecting decreased microbial diversity and ecological imbalance (79). Characteristic shifts include a decline in Firmicutes abundance with concomitant increases in Bacteroidetes and Proteobacteria. The expansion of pro-inflammatory taxa can elevate circulating lipopolysaccharide (LPS), promoting systemic inflammation and potentially accelerating PD pathology (80, 81). These alterations may compromise intestinal barrier function, thereby promoting gut inflammation and systemic inflammatory responses that ultimately affect the CNS via the gut-brain axis (82). Indirectly, they regulate BBB permeability and neuroinflammatory responses, thereby affecting neuronal survival. For instance, probiotic supplementation with *Bifidobacterium lactis* MH-022 has been shown to improve motor function and neuronal integrity in PD models by enhancing antioxidant defenses and suppressing neuroinflammation (83). A central pathological feature of PD is the aggregation of α-synuclein. Gut dysbiosis may promote the misfolding and aggregation of α-synuclein in the ENS (84, 85). These pathological forms can potentially propagate to the CNS via the vagus nerve, leading to Lewy body formation and PD-related neurodegeneration (86, 87).

Microbial metabolites-including SCFAs, H_2_S, and tryptophan derivatives-also contribute to PD progression. SCFAs exhibit anti-inflammatory and neuroprotective effects; for instance, propionic acid exerts neuroprotection by activating the FFAR3 receptor, alleviating motor dysfunction and dopaminergic neuron loss in PD models (88). However, their levels are significantly reduced in PD patients, potentially diminishing their inhibitory effect on neuroinflammation (89). Other microbial metabolites, including H_2_S, Adenosine (ADO) and Quinolinic acid (QA) may promote oxidative stress and mitochondrial dysfunction, exacerbating neuronal injury (90–95).

Immune regulation represents another key mechanism by which the gut microbiota influences PD. Research indicates that in PD, the gut microbiota affects immune responses and neuronal function by regulating amino acid metabolism (e.g., branched-chain amino acids and aromatic amino acids) (96). Alterations in microbial composition can activate innate and adaptive immunity, leading to sustained neuroinflammation and neuronal damage (83). For example, certain gut bacteria (e.g., Ruminococcaceae) can enhance immune responses by activating dendritic cells (DCs) and CD8^+^ T cells (97). Furthermore, dysbiosis may promote α-synuclein aggregation and propagation by modulating Toll-like receptor (TLR) signaling pathways (98).

### 3.3 Amyotrophic lateral sclerosis

ALS is a progressive NDDs characterized by the selective loss of motor neurons. Its pathogenesis is multifactorial, involving a complex interplay of genetic, epigenetic, and environmental influences (99). In recent years, the gut microbiota has emerged as a key modulator in ALS pathophysiology, primarily through its role in the bidirectional GBA that links the gastrointestinal tract with the CNS (100).

Patients with ALS exhibit decreased gut microbial diversity and significant compositional shifts, including an increased abundance of Enterobacteriaceae and reduced levels of beneficial genera such as *Bifidobacterium* and *Clostridium sensu stricto (*101–103). This dysbiotic state can trigger non-cell-autonomous neuroinflammation by activating microglia and astrocytes, thereby accelerating motor neuron injury (99). Furthermore, impaired intestinal barrier integrity-driven by microbial imbalance-facilitates the systemic translocation of bacterial metabolites and pro-inflammatory mediators, which may subsequently compromise CNS immune homeostasis (104).

Notably, microbial metabolites play a dual role in ALS progression. SCFAs such as butyrate exert anti-inflammatory and neuroprotective effects, yet their levels are often diminished in ALS patients (105). Similarly, SCFAs can directly stimulate the vagus nerve, thereby regulating inflammatory responses and neuroprotective mechanisms within the CNS (106). The gut microbiota further influences ALS by shaping peripheral immune responses. Dysregulation promotes the infiltration and skewed differentiation of T cell populations-such as an imbalance between Th17 and Treg cells-which amplifies neuroinflammation and motor neuron damage (99).

Metagenomic analyses also reveal marked alterations in microbial metabolic pathways in ALS, particularly those involved in neurotransmitter synthesis and neuroprotection (107). For instance, tryptophan metabolites produced by the gut microbiota-such as serotonin and kynurenine-exhibit dysregulation in ALS patients, with elevated kynurenine levels closely linked to neuroinflammation and oxidative stress (108).

In summary, the gut microbiota contributes to NDDs pathogenesis through metabolite signaling, immune modulation, and gut-brain communication. However, given the rapid progression and heterogeneity of NDDs, the precise causal role of microbial communities remains incompletely defined. Future studies should focus on elucidating the specific mechanisms underlying gut-brain interactions in NDDs and evaluating the translational potential of microbiota-targeted interventions.

## 4 Therapeutic measures based on gut microbiota

Based on the cognitive advancement that gut microbiota influences NDDs via the MGBA, the treatment regimens for NDDs have gradually broadened from single neural therapy to the regulation of gut microbiota. Probiotics (beneficial live microorganisms) and prebiotics (dietary components that stimulate the growth of beneficial bacteria) for regulating the intestinal flora have emerged as a highly promising novel treatment and prevention strategy (109, 110). Specific strains, including Lactobacillus and Bifidobacterium, have the potential to restore the equilibrium of the intestinal flora, decrease the concentrations of pro - inflammatory factors (e.g., IL - 6, TNF - α), and enhance the expression of brain - derived neurotrophic factor (BDNF), consequently improving cognitive function (19, 111). Clinical trials have demonstrated that probiotic mixtures can notably delay cognitive decline in patients with AD (111).

Probiotics, including Lactobacillus and Bifidobacterium, regulate the MGBA via multiple pathways. Initially, they are capable of restoring the equilibrium of the intestinal flora, curbing the excessive proliferation of pathogenic bacteria such as Akkermansia (whose population is significantly elevated in patients with AD and PD), and augmenting the population of SCFAs - producing bacteria (e.g., Faecalibacterium). These SCFAs - producing bacteria can inhibit the over - activation of microglia through the BBB and mitigate neuroinflammation (112, 113). Secondly, probiotics directly secrete neuroactive substances (e.g., GABA, 5 - hydroxytryptamine) and influence brain function through the vagus nerve or the circulatory system (114). For example, clinical research has demonstrated that supplementation with Lactobacillus rhamnosus can notably reduce Aβ plaque deposition in the hippocampus of AD model mice and enhance the phagocytic activity of microglia (115). Moreover, probiotics can also fortify the tight junctions of the intestinal epithelium, diminish the entry of endotoxins (such as LPS) into the bloodstream, thus reducing the damage to the BBB induced by systemic inflammation (20).

Prebiotics, classified as indigestible dietary fibers (e.g., inulin and fructooligosaccharides), selectively stimulate the proliferation of probiotics and produce SCFAs (e.g., butyric acid and propionic acid) via fermentation (116). A randomized controlled trial conducted on patients with PD indicated that prebiotic intervention notably elevated the concentration of butyric acid in feces and enhanced motor symptom scores (Unified Parkinson’s Disease Rating Scale, UPDRS). The effect was positively associated with the abundance of butyrate - producing bacteria, such as Roseburia, in the gut microbiota (117). Moreover, prebiotics can modulate the tryptophan metabolic pathway, mitigate the accumulation of neurotoxic metabolites (e.g., QA), and augment the levels of 5 - hydroxytryptamine precursors, thereby indirectly alleviating depression and cognitive impairment (118). The combined utilization of probiotics and prebiotics (synbiotics) demonstrates a synergistic effect. For example, in an ALS model, synbiotic intervention not only rectified gut microbiota dysbiosis but also postponed motor neuron degeneration by reducing plasma interleukin - 6 (IL - 6) and tumor necrosis factor - α (TNF - α) levels (119).

Fecal microbiota transplantation (FMT): The transplantation of intestinal microbiota from healthy donors to patients suffering from NDDs can reshape their microbial composition and mitigate the deposition of pathogenic proteins. For example, subsequent to FMT, patients diagnosed with PD have demonstrated enhancements in both motor symptoms and intestinal permeability (120, 121). In PD models, the transplantation of microbiota from healthy donors can substantially reverse motor deficits induced by MPTP in mice, suppress microglial/astrocyte activation in the substantia nigra - striatum region, and alleviate mitochondrial oxidative stress via the AMPK/SOD2 pathway (122). Clinical investigations have further verified that following FMT, PD patients exhibit ameliorated constipation symptoms and a reduced intestinal transit time, also display notable improvements in subjective motor and non - motor symptoms, such as cognitive function (123).

## 5 Discussion and conclusion

In recent years, the mechanism by which gut microbiota influence NDDs via the gut-brain axis has garnered widespread attention. This paradigm underscores that the pathogenesis of NDDs extends beyond the brain, with gut microbes modulating CNS function through metabolite secretion, neurotransmitter synthesis, and immunomodulatory signaling (124). Microbial metabolites-such as SCFAs-participate in NDDs pathology by regulating the function of microglia, astrocytes, and oligodendrocytes (16). Additionally, gut bacteria produce neurotransmitters (e.g., serotonin, GABA) and gut hormones (e.g., glucagon-like peptide-1), which transit the MGBA to influence neuronal excitability and synaptic plasticity, thereby shaping disease progression in AD and other NDDs (6, 7, 125, 126).

The immune system represents another critical mediator through which gut microbes participate in NDDs. Dysbiosis can compromise both intestinal and BBB integrity, facilitating the translocation of microbial components and inflammatory mediators into the CNS. This process exacerbates neuroinflammation and accelerates neuronal injury (104, 127, 128).

Despite substantial advances in delineating the role of the MGBA in NDDs, important limitations remain. Current evidence is largely correlative, and definitive causal links have yet to be fully established (16). Moreover, the gut microbiota exhibits considerable interindividual variation in composition and functional output, which challenges the generalizability and reproducibility of findings across studies (7, 9, 104, 129). Future research should prioritize longitudinal and interventional studies to clarify causative mechanisms, alongside efforts to standardize methodologies and account for host confounding factors.

Building on advances in MGBA research, gut microbes and their metabolites have emerged as promising targets for diagnosing and treating NDDs. Therapeutic strategies such as probiotics, prebiotics, dietary interventions, and FMT have shown encouraging results in both preclinical and clinical settings (**Table 1**). For instance, probiotic interventions (e.g., *Lactobacillus plantarum* and *Bifidobacterium breve*) can restore gut microbial balance, also improve cognitive function and neuroinflammation by modulating gut-brain axis signaling pathways (177). Another notable example is GV-971, a recently developed oligosaccharide derived from marine algae, which has been shown to significantly improve cognitive function in patients with mild-to-moderate AD by targeting gut microbiota dysbiosis and dampening neuroinflammation (178).

**Table 1:** Clinical studies on the role of microbiota-gut-brain axis (MGBA) disruption in neurodegenerative diseases.

Treatment	NCT number	Brief summary	Conditions	Interventions	Sex	Age	Phases	Enrollment	References
Prebiotics	NCT06948929	A new synbiotic formula (SCV09)	AD	DIETARY_SUPPLEMENT: SCV09	ALL	Adult, Older Adult	NA	30	(130)
	NCT04512599	A dietary bar	PD	OTHER: Prebiotic Bar	ALL	Child, Adult, Older Adult	NA	20	(131)
	NCT05576818	Lactobacillus acidophilus probiotic with prebiotic fibers as an adjuvant therapy	PD	DRUG: Lactobacillus acidophilus 10 billion colony forming unit (CFU) and prebiotic fibers	ALL	Adult, Older Adult	PHASE3	66	(132)
	NCT07127120	A prebiotic fiber blend	PD	DIETARY_SUPPLEMENT: Prebiotic fiber blend	ALL	Adult, Older Adult	NA	20	(133)
	NCT04451096	Multi-strain probiotic (Lactobacillus spp and Bifidobacterium spp at 30 X 109 CFU) with fructo-oligosaccaride (FOS) or placebo (fermented milk)	PD	DIETARY_SUPPLEMENT: Probiotics with prebiotic; DIETARY_SUPPLEMENT: Placebo	ALL	Adult, Older Adult	PHASE3	48	(134)
Probiotics	NCT05145881	Five strains of probiotics (Bifidobacterium breve Bv-889, B. Longum subspecies infantis BLI-02, B. Bifidum VDD088, B. Animalis subsp. Lactis CP-9, Lactobacillus plantarum PL-02) with anti-oxidant and anti-inflammatory functions.	AD	DIETARY_SUPPLEMENT: Low dose probiotics; DIETARY_SUPPLEMENT: Normal dose probiotics	ALL	Adult, Older Adult	NA	40	(135)
	NCT06019117	A probiotic preparation (Probiotic K10)	PD; AD	DIETARY_SUPPLEMENT: Probiotic K10; DRUG: Placebo	ALL	Adult, Older Adult	NA	104	(136)
	NCT06181513	Probiotics	Neurodegenerative Diseases	DRUG: Probiotic Blend Capsule	ALL	Older Adult	EARLY_PHASE1	40	(137)
	NCT05521477	A probiotic (SLAB51)	AD	DIETARY_SUPPLEMENT: SLAB51	ALL	Adult, Older Adult	NA	3	(138)
	NCT06948929	A new synbiotic formula (SCV09)	AD	DIETARY_SUPPLEMENT: SCV09	ALL	Adult, Older Adult	NA	30	(130)
	NCT03991195	The probiotic supplemented group with amci will take certain Bifidobacterium	AD	DIETARY_SUPPLEMENT: Probiotic supplemented intervention; DIETARY_SUPPLEMENT: Placebo	ALL	Adult, Older Adult	NA	90	(139)
	NCT05173701	The investigators developed a clinical trial protocol for the evaluation of probiotics' effects on the peripheral immune system profile in PD patients.	PD	DIETARY_SUPPLEMENT: Probiotics; DIETARY_SUPPLEMENT: Placebo	ALL	Child, Adult, Older Adult	NA	88	(140)
	NCT05576818	This study aims to investigate the possible efficacy and safety of synbiotic preparation of Lactobacillus acidophilus probiotic with prebiotic fibers as an adjuvant therapy in the treatment of PD	PD	DRUG: Lactobacillus acidophilus 10 billion colony forming unit (CFU) and prebiotic fibers	ALL	Adult, Older Adult	PHASE3	66	(132)
	NCT05568498	This study evaluates the use of an oral multi-strain probiotic in the treatment of depression in individuals with PD.	PD	DIETARY_SUPPLEMENT: Probiotic; DIETARY_SUPPLEMENT: Placebo	ALL	Adult, Older Adult	PHASE2	60	(141)
	NCT03968133	This study evaluates the use of an oral multi-strain probiotic in the treatment of anxiety in individuals with PD.	PD	DIETARY_SUPPLEMENT: Probiotic; DIETARY_SUPPLEMENT: Placebo	ALL	Adult, Older Adult	PHASE2	61	(142)
	NCT05146921	This is an exploratory study investigating the effects probiotic intervention (Symprove) on gut and general health in 60 patients with PD and constipation.	PD	DIETARY_SUPPLEMENT: Multi-strain probiotic; OTHER: Placebo	ALL	Adult, Older Adult	NA	60	(143)
	NCT03377322	This is a trial to evaluate the efficacy of probiotics in the treatment of constipation in PD.	PD	DRUG: Probiotic Capsule; DRUG: Placebo Capsule	ALL	Adult, Older Adult	NA	72	(144)
	NCT04871464	The improvement effect of bifidobacterium triad live bacteria capsules (BIFICO) on the motor symptoms, constipation and sleep conditions of patients with mild to moderate PD	PD	DRUG: Live Combined Bifidobacterium, Lactobacillus and Enterococcus Capsules; OTHER: Placebo	ALL	Adult, Older Adult	PHASE4	240	(145)
	NCT06487975	The aim of this study is to evaluate whether the administration of Bacillus Subtilis influences gut and blood biomarkers relevant to the proposed mechanism(s) of action, as well as being acceptable as a regular supplement for people with PD.	PD	DIETARY_SUPPLEMENT: Bacillus Subtilis; OTHER: Placebo	ALL	Adult, Older Adult	NA	48	(146)
	NCT04722211	This RCT study is designed to examine the extent to which L. Plantarum PS128 can improve symptoms in PD patients.	PD	DIETARY_SUPPLEMENT: PS128; DIETARY_SUPPLEMENT: placebo	ALL	Adult, Older Adult	NA	120	(147)
	NCT04293159	The aim of the study is to collect data for the assessment of the Lactobacillus casei DG (Enterolactis ®duo) effect on constipation and on neuropsychological performance.	PD	DIETARY_SUPPLEMENT: Lactobacillus casei DG (Enterolactis ®duo)	ALL	Adult, Older Adult	NA	30	(148)
	NCT06548256	This is a study to assess the efficacy of Clostridium Butyricum Miyairi on the motor and non-motor symptoms of PD.	PD	DRUG: Miyarisan-BM (Clostridium Butyricum Miyairi)	ALL	Adult, Older Adult	NA	400	(149)
	NCT03324399	New Biotic, LLC has submitted an Orphan Drug Designation Application for an investigational probiotic.	ALS	DIETARY_SUPPLEMENT: probiotic	ALL	Adult, Older Adult	NA	5	(150)
	NCT06051123	The aim of this study is to assess the impact of a probiotic formulation on participants with ALS-FTDSD.	ALSFTD	DIETARY_SUPPLEMENT: Probiotic; DIETARY_SUPPLEMENT: Placebo	ALL	Adult, Older Adult	NA	150	(151)
Dietary intervention	NCT03691519	The aim is to evaluate the efficacy of omega-3(DHA+EPA) supplementation on cognitive decline in older adults with low DHA/EPA status and subjective memory complaints or family history of AD.	AD	DRUG: Omega-3 treatment; DIETARY_SUPPLEMENT: Placebo	ALL	Older Adult	PHASE3	774	(152)
	NCT06705517	This study assesses whether an MD can improve motor and non-motor symptoms in PD patients.	PD	BEHAVIORAL: Mediterranean Diet	ALL	Adult, Older Adult	NA	44	(153)
	NCT02274324	To evaluate the role of dietary modifications of 3 different diets on clinical outcomes in patients with PD.	PD	DIETARY_SUPPLEMENT: different diets	ALL	Adult, Older Adult	NA	20	(154)
	NCT03851861	Mediterranean diet	PD	OTHER: Mediterranean Diet	ALL	Adult, Older Adult	NA	8	(155)
	NCT07213856	The goal of this clinical trial is to evaluate whether a dietitian-guided nutritional intervention can improve constipation symptoms in people with PD.	Parkinsons Disease	BEHAVIORAL: Dietitian-Guided Nutritional Intervention	ALL	Adult, Older Adult	NA	54	(156)
	NCT05469997	This study aims to investigate the safety of modified Mediterranean-ketogenic interventions, as it relates to the gut microbiome health in patients with PD.	PD	BEHAVIORAL: Mediterranean-Ketogenic Diet; DIETARY_SUPPLEMENT: Mediterranean diet supplemented with medium-chain triglyceride oil	ALL	Adult, Older Adult	NA	50	(157)
	NCT00906763	This study aims to investigate the effects of high cocoa content (85%) dark chocolate and chocolate without any cocoa components (white chocolate) on the motor symptoms of PD patients (measured through the third part of the UPDRS (motor score)).	PD	DIETARY_SUPPLEMENT: Chocolate	ALL	Adult, Older Adult	NA	23	(158)
	NCT06207136	The goal of this pilot study is to examine the feasibility and effects of an 18-month intervention diet compared to an active control diet (standard diet) in those living with PD, without dementia.	PD	OTHER: Mediterranean-style diet	ALL	Adult, Older Adult	NA	40	(159)
	NCT04683900	The aim is to explore the impact of Mediterranean diet intervention on the gastrointestinal function of patients with PD.	PD	OTHER: Standard of care + Mediterranean diet (intervention); OTHER: Standard of care (control)	ALL	Adult, Older Adult	NA	46	(160)
	NCT06463769	Compared with the ordinary German diet, the "new Nordic oligosaccharide diet" (a culturally adapted diet rich in fermentable fibers and phytochemicals) will have a beneficial effect on the intestinal microbiota of patients with PD.	PD	BEHAVIORAL: predominantly plant-based New Nordic LPF-diet program	ALL	Adult, Older Adult	NA	75	(161)
	NCT07178067	This observational study explored the connection between the gut microbiota and the brain in patients with ALS, specifically the modulation of short-chain fatty acids during disease progression and after following a Mediterranean diet for 6 months.	ALS	OTHER: Mediterranean diet	ALL	Adult, Older Adult		44	(162)
	NCT04172792	The investigators seek to investigate whether an ultra-high caloric diet (UHCD), featuring the double amount of calories compared to LIPCAL-ALS, will be well tolerated by ALS patients and may serve as an intervention for a potential LIPCALII study.	ALS	DIETARY_SUPPLEMENT: high-caloric fatty diet; DIETARY_SUPPLEMENT: ultra-high-caloric fatty diet; DIETARY_SUPPLEMENT: ultra-high-caloric carbohydrate-rich diet	ALL	Adult, Older Adult	PHASE1	64	(163)
	NCT02306590	This is a trial comparing placebo with high caloric fatty diet for drinking as add-on therapy to 100 mg RILUZOLE in ALS in 200 enrolled patients.	ALS	DIETARY_SUPPLEMENT: Calogen; DIETARY_SUPPLEMENT: Placebo	ALL	Adult, Older Adult	NA	207	(164)
	NCT02152449	The aim of this study is to determine whether early oral nutritional supplementation (ONS) is effective in treating patients with ALS.	ALS	DIETARY_SUPPLEMENT: Oral nutritional supplementation	ALL	Adult, Older Adult	NA	229	(165)
Fecal Microbiota Transplant (FMT)	NCT03998423	The goal of this study is to assess the safety and feasibility of an oral FMT intervention for AD.	AD	BIOLOGICAL: Fecal Microbiota Transplant	ALL	Adult, Older Adult	PHASE1	5	(166)
	NCT06920212	To investigate the clinical safety and efficacy of FMT in AD patients, as well as the changes in the gut microbiota of AD patients before and after FMT.	AD	PROCEDURE: FMT capsule	ALL	Adult, Older Adult	NA	30	(167)
	NCT04854291	Patients with PD receive treatment through fecal microbiota transplantation from donors or by directly infusing their own feces into the rectum.	PD	OTHER: Administration of donor FMT; OTHER: Administration of placebo	ALL	Adult, Older Adult	NA	51	(168)
	NCT06388863	Patients undergoing FMT intervention	PD	DRUG: Healthy donor-derived FMT capsule; DRUG: Placebo capsule	ALL	Adult, Older Adult	NA	76	(169)
	NCT03808389	Determine the impact of FMT on the symptoms and progression of PD in patients.	PD	OTHER: Donor FM; OTHER: Autologous FMT	ALL	Adult, Older Adult	NA	49	(170)
	NCT03876327	This study aims to further explore the potential application of fecal microbiota transplantation in treating constipation and possibly improving the motor symptoms of patients with PD.	PD	PROCEDURE: FMT	ALL	Adult, Older Adult	PHASE2|PHASE3	10	(171)
	NCT07038226	The aim of this clinical trial is to assess the impact of FMT delivered via oral capsules in patients with PD who suffer from refractory constipation.	PD	BIOLOGICAL: Placebo; BIOLOGICAL: Oral capsule-delivered FMT	ALL	Adult, Older Adult	NA	16	(172)
	NCT06647277	The study investigated the effect of FMT on reducing the severity of symptoms in patients with PD.	PD	COMBINATION_PRODUCT: FMT	ALL	Adult, Older Adult	PHASE1	10	(173)
	NCT04837313	This study aims to evaluate the efficacy and safety of fecal microbiota transplantation in treating constipation symptoms in PD patients who are taking a stable dose of levodopa.	PD	PROCEDURE: FMT	ALL	Adult, Older Adult	NA	30	(174)
	NCT03766321	This study aims to evaluate the effects of FMT on patients with ALS in terms of biology and disease improvement.	ALS	BIOLOGICAL: FMT; BIOLOGICAL: Placebo	ALL	Adult, Older Adult	NA	42	(175)
	NCT07017946	A study on intestinal microbiota transplantation for patients with ALS.	ALS	DRUG: MTP-101C	ALL	Adult, Older Adult	PHASE1|PHASE2	20	(176)

Nevertheless, most clinical studies conducted to date are limited by small sample sizes, and there remains a need for standardized intervention protocols as well as longer-term efficacy evaluations. The long - term safety of microbiota transplantation, the optimal donor screening criteria, and the dosage regimens remain to be clarified (179). The integration of multi-omics approaches with artificial intelligence-based analytics holds considerable potential to elucidate the gut microbiota’s role in NDDs more systematically and to guide the development of targeted therapeutic interventions.

In summary, the MGBA plays a critical role in the initiation and progression of NDDs, functionally linking central pathology to peripheral metabolic and immune processes. Understanding the involvement of gut microbes in NDDs provides novel mechanistic insights into their complex etiology and opens new avenues for therapeutic innovation. Future studies should focus on delineating the precise causal mechanisms underlying gut-brain communication and refining microbiota-based strategies to facilitate their successful translation into clinical practice.
